# Reorganization and functional divergence of the CD4+ memory T cell compartment in hidradenitis suppurativa

**DOI:** 10.3389/fimmu.2026.1831664

**Published:** 2026-07-14

**Authors:** Laura Casals-Diaz, Jorge Romaní, Abir Ezzaanouni, Madalina Raducu, Cristina Vila, Lluís Boix, Amadeu Gavaldà, Antonio Guilabert

**Affiliations:** 1 Disease Biology Department, Almirall R&D Center, Sant Feliu de Llobregat, Barcelona, Spain; 2 Hospital General de Granollers, Dermatology, Granollers, Barcelona, Spain; 3 Pathobiology of Vascular Tumours and Malformations, Institut de Recerca Sant Joan de Déu, Pediatric Cancer Center Barcelona, Esplugues de Llobregat, Barcelona, Spain; 4 Universitat Internacional de Catalunya, Barcelona, Spain

**Keywords:** CCR7, central memory T cells, hidradenitis suppurativa, resident memory T cells, Th17

## Abstract

Hidradenitis suppurativa (HS) is a chronic inflammatory skin disease with poorly understood and complex pathophysiology with interplay between many immune and stromal cells. While the T cell infiltrate in HS is well-documented, the relationship between systemic memory T cells and those within the cutaneous microenvironment has yet to be fully elucidated. We performed a comparative analysis of skin and peripheral blood mononuclear cells (PBMCs) samples from HS patients and healthy controls using flow cytometry to evaluate T cell subsets and their cytokine profile with focus on TNF-α, IFN-γ, IL-17A. Within the memory compartment, CD4+ central memory T cells (T_CM_) were profoundly expanded in HS lesions (21%) compared to healthy skin (<1%). A positive correlation was observed between the levels of skin-infiltrating T_CM_ and those found in the systemic circulation. Conversely, CD4+ resident memory T cells (T_RM_) were significantly depleted in HS lesions (~30% to <10%). Functional assays revealed a marked discordance between tissues; skin-derived memory cells exhibited a hyper-responsive phenotype, producing significantly higher levels of TNF-α, IFN-γ, and IL-17A than matched PBMCs. These data suggest that HS is characterized by a synchronized peripheral-lesional axis of CD4+ T_CM_ cells. The T_RM_ deficit may reflect phenotypic plasticity or retrograde migration during chronic inflammation. Crucially, the HS microenvironment appears to reprogram recruited memory cells, leading to a tissue-specific functional activation not reflected in the systemic circulation.

## 1 Introduction

Hidradenitis suppurativa (HS) is a debilitating chronic inflammatory skin disease characterized by painful, deep-seated nodules, abscesses, and epithelialized tunnels, primarily affecting intertriginous areas (1, 2). Typically emerging in early adulthood, HS carries a profound burden on patient quality of life and is frequently associated with systemic comorbidities, including metabolic syndrome and inflammatory arthritis, reflecting a state of widespread systemic inflammation. The pathogenesis of HS is multifactorial, involving a complex interplay between innate and adaptive immunity. Key drivers include the Th17/Th1 axis (3), with significant contributions from macrophages, neutrophils, and IL-17A-producing T cells (4). In advanced stages, chronic inflammation leads to irreversible tissue remodeling and the formation of tertiary lymphoid structures (TLS) within the dermis (5). While the introduction of biologics targeting TNF-α [adalimumab (6),] and IL-17 [secukinumab, bimekizumab (7, 8)] has improved management, a substantial number of patients remain non-responders, highlighting the need for a deeper understanding of the cellular dynamics and cell repertoire driving disease persistence. Despite the well-documented T cell infiltrate within HS lesions, the relationship between these cutaneous populations and the systemic immune compartment remains poorly defined. It is unclear whether the expanded memory T cell pools in the skin are maintained through local proliferation or via the continuous recruitment of circulating precursors. Furthermore, whether systemic T cell frequencies can serve as reliable surrogates for the cutaneous inflammatory state remains a subject of debate (9). In this study, we aimed to characterize the peripheral-lesional axis of memory T cells in HS. By performing a comparative analysis of matched skin and peripheral blood mononuclear cells (PBMCs), we sought to define the numerical and functional synchronization between these compartments, focusing on the distinct roles of central memory (T_CM_) and resident memory (T_RM_) T cell subsets.

## 2 Ethics statement

All experiments performed on human samples were conducted in accordance with the Declaration of Helsinki. Healthy human blood was obtained from donors from the Hospital de la Santa Creu i Sant Pau (Barcelona) and approved by its medical ethics committee (number EC/20/345/6135). Healthy skin samples were obtained from plastic reduction surgery at Clinica Planas (Barcelona), and approved by the ethics committee of Bellvitge Hospital Universitari (code PR198/22). Hidradenitis suppurativa skin and blood samples were obtained from a monographic HS clinic at Hospital de Granollers; the study protocol was approved by the ethics committee of the hospital, with CEIm code 20222048. Written informed consent was obtained from all donors and patients.

## 3 Methods

### 3.1 Samples

HS skin and matched blood samples (*n* = 7) were collected from April to December 2024 following the inclusion criteria: adult patients (>18) with Hurley stage II or III HS, candidates for surgical excision of a lesional area. Blood samples were drawn from venous access following current clinical practice. Exclusion criteria included any treatment with antibiotics, immunosuppressive, or immunomodulatory therapies (including biologics) within 2 months prior to surgery, as well as enrollment in ongoing clinical trials. Patient demographic and clinical characteristics for the HS cohort are detailed in **Supplementary Table 1**.

Healthy skin samples (*n* = 5) were obtained from the submammary and abdominal waste material of women who underwent breast or abdominal reduction surgery (regions suitable as a control sample because the inframammary and abdominal folds are common sites for HS lesions). All samples were sent to our laboratory by courier during the first two hours after surgical dissection. Additionally, peripheral blood samples were obtained from healthy volunteers (*n* = 8) to serve as systemic controls.

Samples were allocated to immunophenotyping and functional assays based on chronological recruitment and cell yield, with specific sample sizes indicated in the figure legends.

### 3.2 Skin and blood sample preparation

Skin samples were processed within early hours after surgery. Briefly, skin samples were rinsed with cold PBS buffer mixed with antibiotic-antimycotic reagent (15240062, Gibco, Life Technologies Europe BV, Bleiswijk, Netherlands), and the subcutaneous fat and hairs carefully removed. Skin was minced into smaller fragments with sterile scissors, digested overnight at 37°C and 5% CO_2_ with 0.8 mg/mL collagenase IV (LS004188, Worthington) and 0.02 mg/mL DNAse I (11284932001, Roche), in RPMI 1640 culture medium (Thermo Fisher) supplemented with 5% human AB serum (Sigma-Aldrich), 42mM HEPES, 100 U/mL penicillin and 100 μg/mL streptomycin, (Sigma, P0781) [protocol based on Du et al., 2021 (10)]. Digestion was terminated by adding PBS with 2mM EDTA, and skin fragments dissociated with gentleMACS C-tubes on a gentleMACS Dissociator (Miltenyi Biotec), washed and filtered through a 70 μm cell strainer (Corning). Red blood cells were lysed with BD FACS™ Lysing Solution (BD Biosciences). Cells were resuspended in cell culture medium, consisting of RPMI-1640 (Sigma, R-8758), 5% human serum (H4522-100ML), 1x GlutaMAX 100x (35050061, Thermo Fisher), 20 mM HEPES (GIBCO, 15630-056) and antibiotics as above.

Peripheral blood mononuclear cells (PBMCs) from blood were isolated with StraightFrom^®^ Whole Blood PBMC Isolation Kit, human (130-126-359, Miltenyi Biotec), and resuspended in the same culture medium as skin cells.

### 3.3 *Ex vivo* stimulation of HS skin cells and PBMCs

Matched skin and blood samples from 4 HS patients were used for evaluation of cytokine production. Single-cell suspensions of HS skin (3 million per well) and PBMCs (1.5 to 2 million per well) were seeded on sterile 12-well polystyrene plates (150628, Nunc) and stimulated o.n, at 37°C 5% CO_2_ with Dynabeads™ Human T-Activator CD3/CD28 (11131D, Gibco, Thermo Fisher Scientific, Vilnius, Lithuania) at a 1:16 (skin) and 1:8 (PBMCs) bead to cell ratio. Protein transport inhibitor cocktail (500x, 00-4980-03, eBioscience, Thermo Fisher), containing brefeldin A and monensin, was added during the last 4h.

### 3.4 Cell surface and intracellular staining for flow cytometry analysis

Cells were centrifuged, washed and stained with Ghost Dye™ Violet 450 (13-0863-T500; Tonbo™ -Cytek^®^), incubated with human Fc Block (564220, BD Biosciences), Brilliant Stain Buffer Plus (566385, BD Biosciences) and True-Stain Monocyte Blocker (426103, Biolegend). Antibodies for extracellular markers were then added and incubated for 20 min in the dark at room temperature. To detect the intracellular production of cytokines, cells were fixed and permeabilized with BD Cytofix/Cytoperm™ Fixation/Permeabilization Kit (554655, BD Biosciences) prior to IL-17A, IFN-γ and TNF-α staining. After washing, cells were analyzed by flow cytometry on a 5-laser Aurora spectral cytometer (Cytek). Cell populations were gated and analyzed with SpectroFlo software (Cytek). Gating strategy is depicted in **Supplementary Figure 1**.

Antibodies used to detect human markers (clone; reference); APC/Fire™ 810 anti-CD45 (HI30; 304076); Brilliant Violet 711™ anti-CD45RA (HI100; 304138); Brilliant Violet 570™ anti-CD45RO (UCHL1; 304226); Alexa Fluor^®^ 647 anti-CD8 (SK1; 344726); FITC anti-CD69 (FN50; 310904); PE/Cyanine7 anti-CD103/Integrin αE (Ber-ACT8; 350212); PerCP/Cyanine5.5 anti-CD197/CCR7 (G043H7; 353220); PE anti-IL-17A (BL168; 512306); Alexa Fluor^®^ 700 anti-TNF-α (MAb11; 502928) and PE/Dazzle™ 594 anti-IFN-γ (B27; 506530), were purchased from Biolegend. Brilliant Ultra Violet™ 395 anti-CD3 (SK7, 363-0036-42, eBioscience™); Brilliant Violet™ 650 anti-CD4 (RPA-T4; 416-0049-42, eBioscience™) and Brilliant Ultra Violet™ 805 anti-CD62L (L-Selectin; DREG-56; 368-0629-42, eBioscience™), purchased from Thermo Fisher.

### 3.5 Statistical analysis

Differences in T cell populations between groups were analyzed using 2-way ANOVA (or mixed-effects model where matched samples were compared) followed by Šídák’s multiple comparisons tests. Pearson correlation coefficient (r) analysis was used to evaluate relationships between skin and blood T cell memory populations. For *ex vivo* cytokine induction assays, 2-way repeated measures ANOVA and Fisher’s LSD test were employed. Differences were considered statistically significant for *P <* 0.05. Data were analyzed and plotted using GraphPad Prism 10.1.2 (GraphPad Software, San Diego, California, USA).

## 4 Results

### 4.1 Characterization of memory cells T cells in healthy and HS skin and peripheral blood

**Figure 1:**
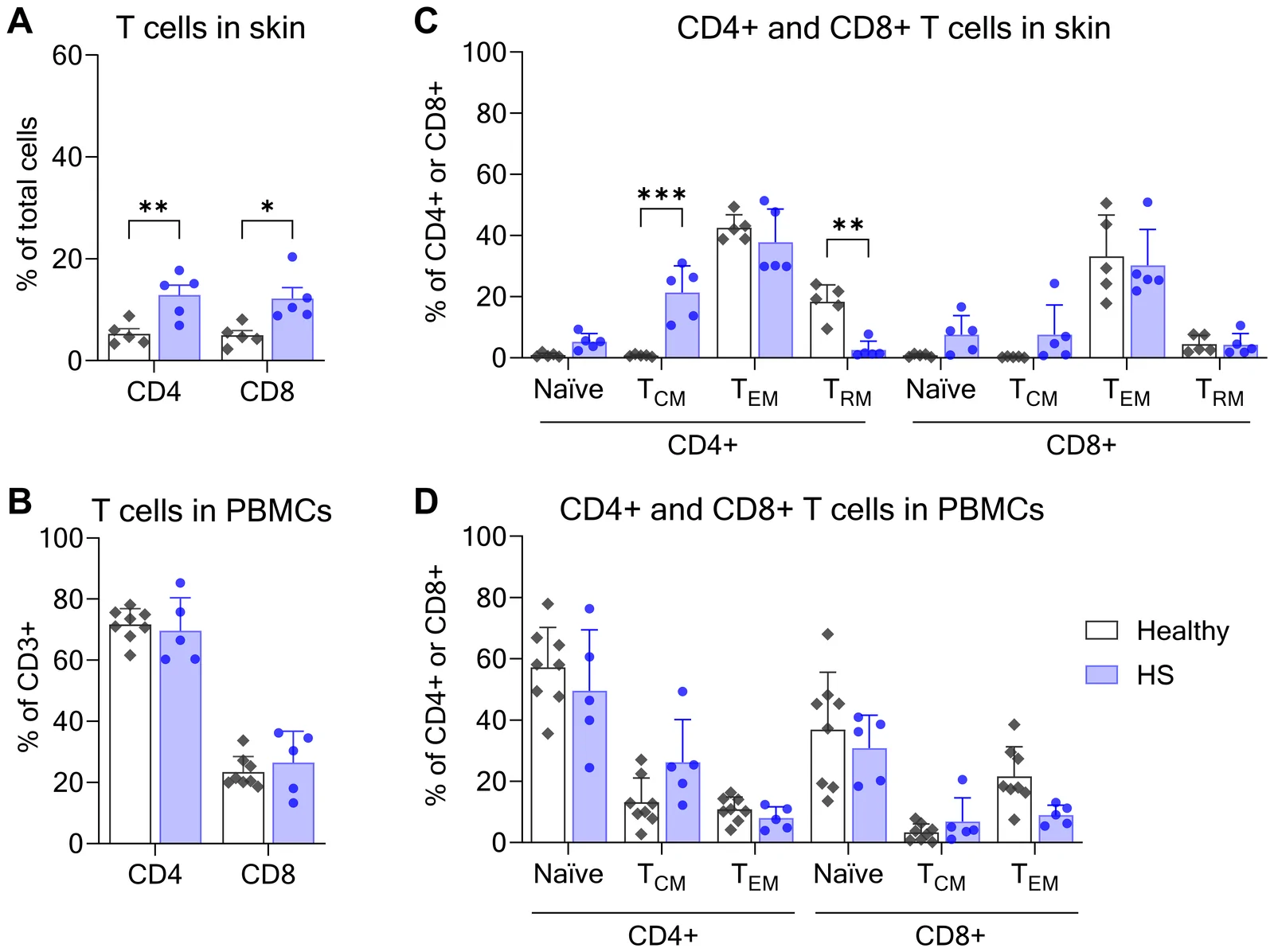
Expansion of central memory T cells (T_CM_) in HS skin. Flow cytometric analysis of total CD4+ and CD8+ T cell subsets in **(A)** the skin and **(B)** peripheral blood. Frequency of naïve and memory subsets within the CD4+ and CD8+ compartments are shown for **(C)** skin and **(D)** peripheral blood. Each dot represents an individual donor (Healthy, diamonds; HS, circles); bars indicate the mean frequency of the population relative to the indicated gate ± standard deviation (SD). Healthy PBMCs, *n* = 8; Healthy skin, *n* = 5; HS skin and PBMCs matched samples (*n* = 5). Detailed gating strategies for T cell memory subsets (Naïve, T_CM_, T_EM_ and T_RM_) are provided in **Supplementary Figure 1**. *P < 0.05, **P < 0.01 and ***P < 0.001 (Two-way ANOVA with Šídák’s multiple comparisons test).

To characterize the immunological landscape of HS, we analyzed the frequency of T cell subpopulations in both the lesional skin and systemic circulation. In the skin, HS patients showed a significant increase in the percentage of both CD4+ (*P <* 0.01) and CD8+ (*P <* 0.05) T cells compared to healthy controls (**Figure 1A**). However, in PBMCs, the percentages of CD4+ and CD8+ cells were similar between both groups (**Figure 1B**).

Analysis of memory differentiation states revealed that the T cell infiltrate in HS skin was primarily composed of effector memory (T_EM_) cells, representing 37.8 ± 10.8% of the CD4+ population and 30.2 ± 11.7% of the CD8+ population (**Figure 1C**). Conversely, naïve T cells were nearly absent in the skin, a finding that stands in sharp contrast to the systemic compartment. In PBMCs, naïve cells remained a major component (49.5 ± 19.9% CD4+ and 30.8 ± 10.7% CD8+), while the frequency of T_EM_ cells was significantly lower than that observed in the skin (7.9 ± 3.7% CD4+, 8.9 ± 3.2%, **Figure 1D**).

The memory profile of HS lesions was further distinguished by a selective enrichment of the CD4+ central memory (T_CM_) subset (**Figure 1C**). While healthy skin maintained a minimal T_CM_ footprint of less than 1%, frequencies in HS skin reached 21.4 ± 8.7% (*P <* 0.001; **Figure 1C**). Interestingly, a strong positive association was observed between CD4+ T_CM_ frequencies in matched skin and PBMC samples from HS patients (Pearson’s r = 0.85). Although this correlation narrowly missed formal statistical significance (P = 0.0676), likely limited by the small cohort of matched pairs available (n = 5), the high correlation coefficient strongly suggests that peripheral blood T_CM_ dynamics tend to mirror the central memory shifts occurring within the cutaneous lesions. Conversely, CD4+ T_EM_ frequencies did not show statistically significant differences between healthy skin (42.4 ± 4.3%) and HS lesions (37.8 ± 10.8%).

We also found a major decrease in the relative frequency of resident memory cells (T_RM_) specifically in the CD4+ group. CD4+ T_RM_ percentages dropped from 18.3 ± 5.6% in healthy skin to 2.6 ± 2.9% in HS (*P <* 0.01; **Figure 1C**). However, the CD8+ T_RM_ population remained stable and did not change between healthy and HS skin.

In contrast to the significant alterations observed in the cutaneous compartment, the memory T cell landscape in the peripheral blood remained largely stable (**Figure 1D**). No significant differences were observed between HS patients and healthy controls, although a non-significant trend toward increased CD4+ T_CM_ frequencies was noted in the systemic circulation of the HS cohort.

### 4.2 Cytokine profile of skin and peripheral blood CD4+ memory T cells

To assess the functional capacity of the expanded memory T cell populations, we compared cytokine production in matched skin and PBMC samples following stimulation with anti-CD3/anti-CD28.

**Figure 2:**
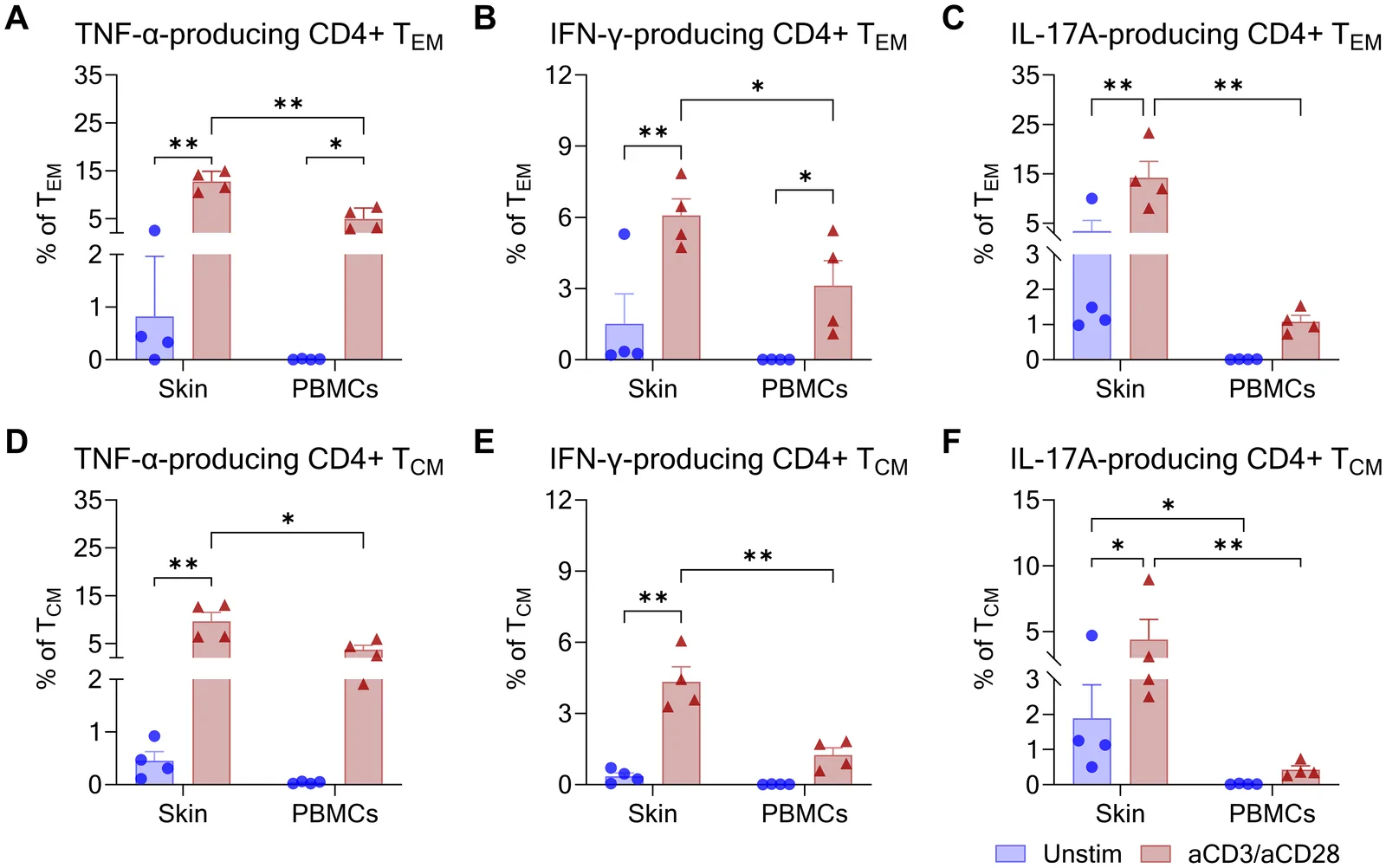
Heightened pro-inflammatory potential of skin *vs* peripheral blood CD4+ memory T cells. Frequencies of cytokine-expressing memory T cell subsets from matched skin and PBMC samples (*n* = 4). Bars represent the mean percentage (± SD) of cytokine-expressing memory T cells *vs* parent, under unstimulated baseline conditions (“Unstim”, blue bars/circles) and following stimulation with anti-CD3/anti-CD28 coated beads (“aCD3/aCD28”, red bars/triangles). Data are shown for TNF-α-producing CD4+ T_EM_ **(A)** and T_CM_ **(D)** cells, IFN-γ-producing CD4+ T_EM_ **(B)** and T_CM_ **(E)**, IL-17A-producing CD4+ T_EM_ **(C)** and T_CM_ **(F)**. Statistical differences between skin and PBMCs and stimulation condition were evaluated by 2-way repeated measures ANOVA and Fisher’s LSD test (*P < 0.05 and **P < 0.01).

While both T_EM_ and T_CM_ subsets from peripheral blood exhibited detectable cytokine induction upon stimulation, the magnitude of this response was significantly lower than that observed in the skin (**Figure 2**). Stimulated skin T_EM_ and T_CM_ cells produced significantly higher levels of TNF-α compared to their PBMCs counterparts (**Figures 2A, D**; *P <* 0.01 and *P <* 0.05, respectively). This tissue-specific hyper-responsiveness was further evidenced by IL-17A (**Figures 2C, F**) and IFN-γ production (**Figures 2B, E**). Notably, IL-17A induction was markedly higher in skin T_EM_ (14.2 ± 6.5%) and T_CM_ (4.4 ± 3.1%) than in stimulated PBMCs, where IL-17A was nearly undetectable (*P <* 0.01 and *P <* 0.05, respectively). While IFN-γ levels were also elevated in skin-derived memory cells compared to peripheral blood, the magnitude of the response was lower than that of TNF-α and IL-17A, peaking at 6.1 ± 1.4% in T_EM_ cells.

In contrast to the potent response observed in the skin, stimulated memory T cells from PBMCs displayed a markedly attenuated cytokine profile. Although stimulation induced detectable increases in TNF-α, IFN-γ, and IL-17A, the percentages of cytokine-positive cells remained significantly lower than those observed in the skin. These results indicate that the memory T cell pool localized within the skin is functionally distinct and hyper-responsive, particularly concerning the TNF-α and IL-17A pathways, which are critical drivers of HS pathogenesis.

## 5 Discussion

Our results reveal a skewed T cell profile in HS. A central finding of this study is the significant enrichment of CD4+ central memory T cells (T_CM_) in HS lesions. While T_CM_ cells are normally rare in healthy skin, they represented 21% of the CD4+ pool in HS patients. Notably, we found a positive correlation between skin and blood T_CM_ frequencies, which suggests that circulating T_CM_ levels accurately reflect the cutaneous memory T cell shifts.

The accumulation of CD45RO+ CCR7+ T_CM_ in HS skin suggests an active recruitment mechanism rather than random infiltration. This recruitment likely occurs within specialized immune hubs, as tertiary lymphoid structures (TLSs). TLSs were identified within severe HS skin lesions (5), in the vicinity of tunnels, and consisted of organized lymphoid aggregates comprising B cells, plasma cells and T cells, which facilitated antigen presentation, B and T cell proliferation, antibody production, and amplification of inflammatory responses. Within these areas, HS fibroblasts expressed high levels of CCL19 and CXCL13 (11), which induced the upregulation of their receptors, CCR7 and CXCR5, on T and B cells, respectively, as well as TNF-α, which further stimulated fibroblasts to produce more chemokines. This signaling feedback loop could promote the continuous trafficking of T_CM_ from the systemic circulation into the skin, where they would contribute to the pathogenic cellular networks found in TLSs. Interestingly, Witte et al. [2023 (9)], who identified transcriptomic changes in blood T helper memory cells that were not mirrored in HS skin lesions, which challenges the notion that systemic memory T cells could be feeding the cutaneous compartment. The correlation we found suggests that while the transcriptional signature might change as cells move from blood to tissue (as seen in our cytokine data), the frequency of the T_CM_ subset remains synchronized between the two compartments. This could indicate that T_CM_ recruitment is a consistent peripheral-cutaneous interface in HS, even if the cells undergo further functional reprogramming upon arrival in the tissue.

A striking finding in our phenotypic analysis of the memory compartment in HS lesions was the significant reduction in the relative frequency of CD4+ T_RM_ cells, compared to healthy skin. This T_RM_ deficit likely stems from the profound follicular destruction and dermal remodeling characteristic of advanced HS (1, 12) which compromises the specialized anatomical niches required for T_RM_ persistence. But also, more interestingly, recent studies have suggested that the tissue residence of T_RM_ cells is reversible. CD4+ CD69+ CD103+ T_RM_ in human skin can down-regulate CD69 and exit the tissue, reenter the bloodstream, and potentially migrate to distant tissue sites (13). It is possible that the observed T_RM_ scarcity is not merely a loss of cells, but rather a phenotypic shift where resident cells downregulate residency markers under the stress of chronic inflammation. These ‘ex-resident’ cells may adopt a T_CM_ phenotype before potentially exiting the tissue. This model of retrograde migration would provide a unified explanation for the simultaneous decrease in T_RM_, and the synchronized increase in T_CM_ across both the cutaneous and systemic compartments.

While the numerical enrichment of T_CM_ is mirrored in the blood, their functional capacity is not. Our stimulated cytokine analysis demonstrates a functional discordance between peripheral and lesional memory cells. CD4+ memory T cells (both T_EM_ and T_CM_) sequestered in HS skin exhibit a significantly higher per-cell potential for TNF-α, IL-17A, and IFN-γ production compared to matched PBMCs. Notably, while systemic T_CM_ remain relatively quiescent, skin-derived T_CM_ appear functionally primed, suggesting that the HS microenvironment provides local signals that lower the activation threshold for pro-inflammatory output. Furthermore, the dominance of TNF-α and IL-17A pathways in our functional assays reinforces the rationale for targeting the Th17/TNF axis in HS, particularly as these cells appear to be hyper-responsive upon arrival in the tissue. In conclusion, our data supports a model where T_CM_ cells serve as a peripheral-cutaneous axis for inflammation, highlighting the importance of targeting both systemic recruitment and local tissue-specific priming in future HS therapies.

## Data Availability

The raw data supporting the conclusions of this article will be made available by the authors, without undue reservation.
