# Correction: Reorganization and functional divergence of the CD4+ memory T cell compartment in hidradenitis suppurativa

**DOI:** 10.3389/fimmu.2026.1940036

**Published:** 2026-08-24

**Authors:** Laura Casals-Diaz, Jorge Romaní, Abir Ezzaanouni, Madalina Raducu, Cristina Vila, Lluís Boix, Amadeu Gavaldà, Antonio Guilabert

**Affiliations:** 1 Disease Biology Department, Almirall R&D Center, Sant Feliu de Llobregat, Barcelona, Spain; 2 Hospital General de Granollers, Dermatology, Granollers, Barcelona, Spain; 3 Pathobiology of Vascular Tumours and Malformations, Institut de Recerca Sant Joan de Déu, Pediatric Cancer Center Barcelona, Esplugues de Llobregat, Barcelona, Spain; 4 Universitat Internacional de Catalunya, Barcelona, Spain

**Keywords:** CCR7, central memory T cells, hidradenitis suppurativa, resident memory T cells, Th17

There were issues with **Figure 1**, **2** and **Supplementary Figure 1** as published. **Figures 1**, **2** lacked the panel labels “A, B, C, etc.”, which makes it difficult to follow the main text and captions. Additionally, we noticed that some details lose sharpness when magnified, which also happened with **Supplementary Figure 1**. In the latter, there was unrelated text on the top right corner “internal use”, which was automatic text that is present in every document of our organization. We have submitted the corrected **Figures 1**, **2** and **Supplementary Figure 1**.

**Figure 1:**
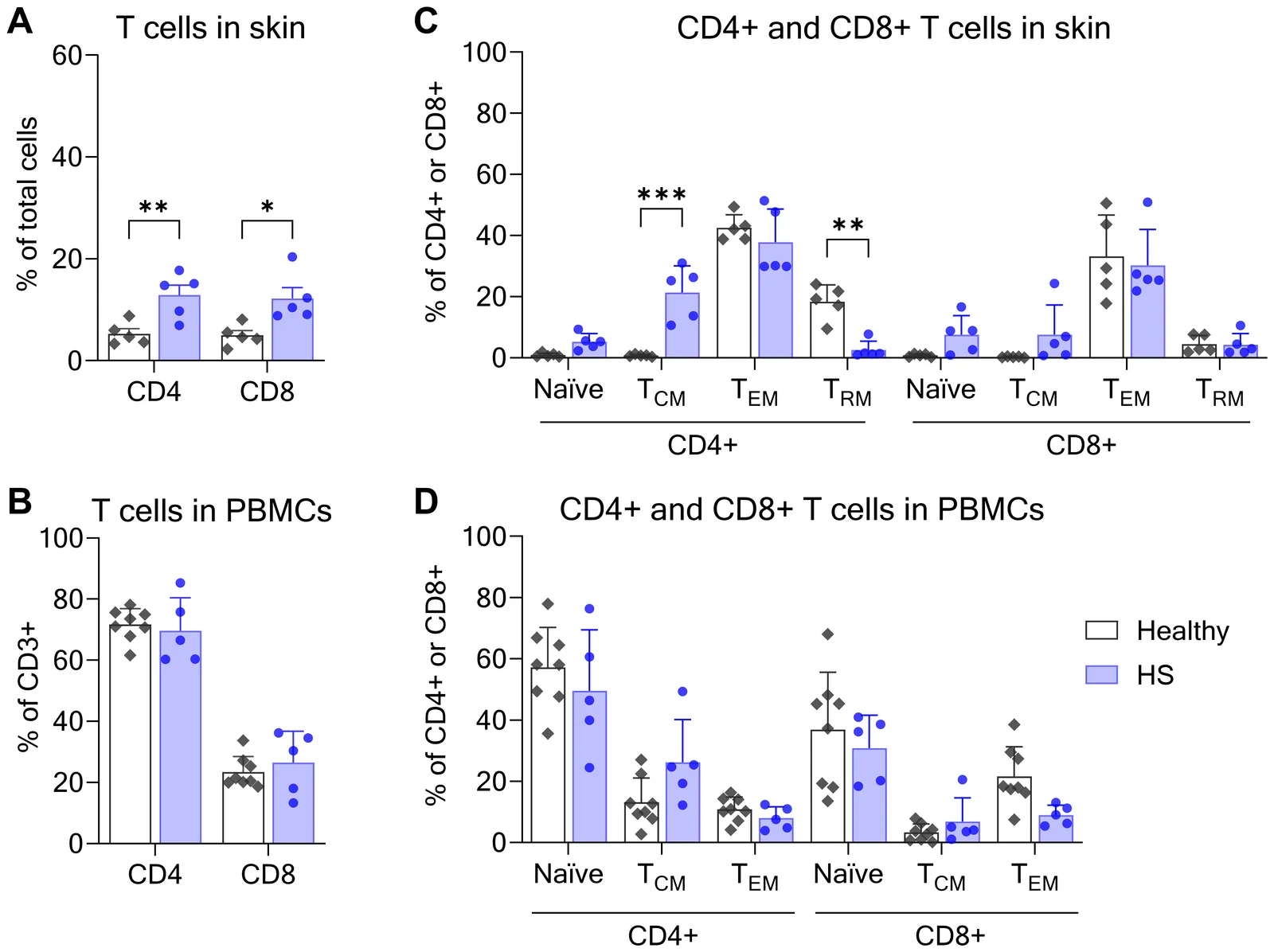
Expansion of central memory T cells (TCM) in HS skin. Flow cytometric analysis of total CD4+ and CD8+ T cell subsets in **(A)** the skin and **(B)** peripheral blood. Frequency of naïve and memory subsets within the CD4+ and CD8+ compartments are shown for **(C)** skin and **(D)** peripheral blood. Each dot represents an individual donor (Healthy, diamonds; HS, circles); bars indicate the mean frequency of the population relative to the indicated gate ± standard deviation (SD). Healthy PBMCs, n = 8; Healthy skin, n = 5; HS skin and PBMCs matched samples (n = 5). Detailed gating strategies for T cell memory subsets (Naïve, TCM, TEM and TRM) are provided in Supplementary Figure 1. *P < 0.05, **P < 0.01 and ***P < 0.001 (Two-way ANOVA with Šídák’s multiple comparisons test).

**Figure 2:**
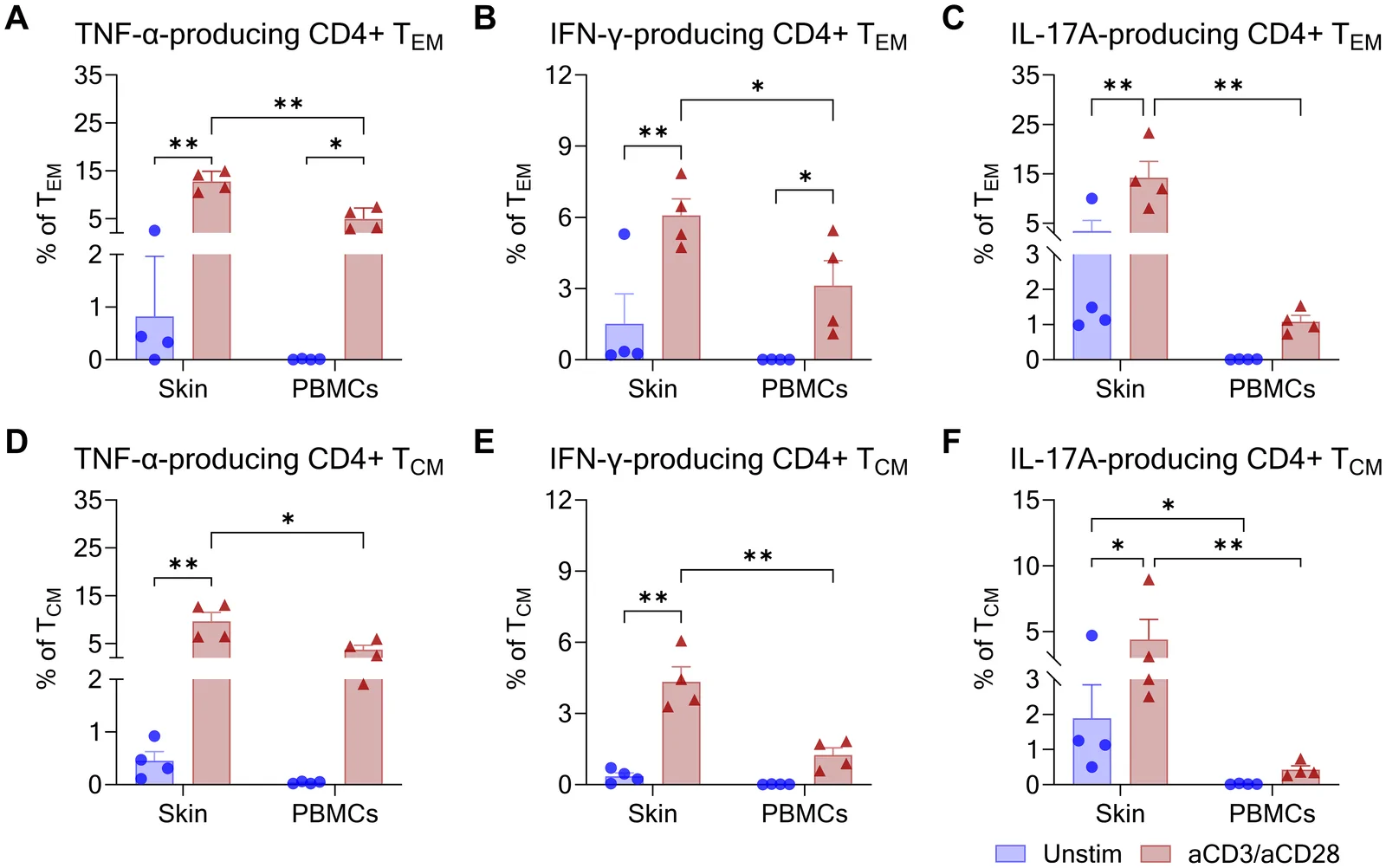
Heightened pro-inflammatory potential of skin vs peripheral blood CD4+ memory T cells. Frequencies of cytokine-expressing memory T cell subsets from matched skin and PBMC samples (n = 4). Bars represent the mean percentage (± SD) of cytokine-expressing memory T cells vs parent, under unstimulated baseline conditions (“Unstim”, blue bars/circles) and following stimulation with anti-CD3/anti-CD28 coated beads (“aCD3/aCD28”, red bars/triangles). Data are shown for TNF-α-producing CD4+ TEM **(A)** and TCM **(D)** cells, IFN-γ-producing CD4+ TEM **(B)** and TCM **(E)**, IL-17A-producing CD4+ TEM **(C)** and TCM **(F)**. Statistical differences between skin and PBMCs and stimulation condition were evaluated by 2-way repeated measures ANOVA and Fisher’s LSD test (*P < 0.05 and **P < 0.01).

The original version of this article has been updated.

