# Supplementary material for: Reorganization and functional divergence of the CD4+ memory T cell compartment in hidradenitis suppurativa
Source: Front Immunol. 2026 Jul 14;17:1831664. doi: 10.3389/fimmu.2026.1831664 (PMC13407087; doi:10.3389/fimmu.2026.1831664)
Supplement: Supplementary file 1 [file DataSheet1.pdf]

INTERNAL USE

Supplementary Figure

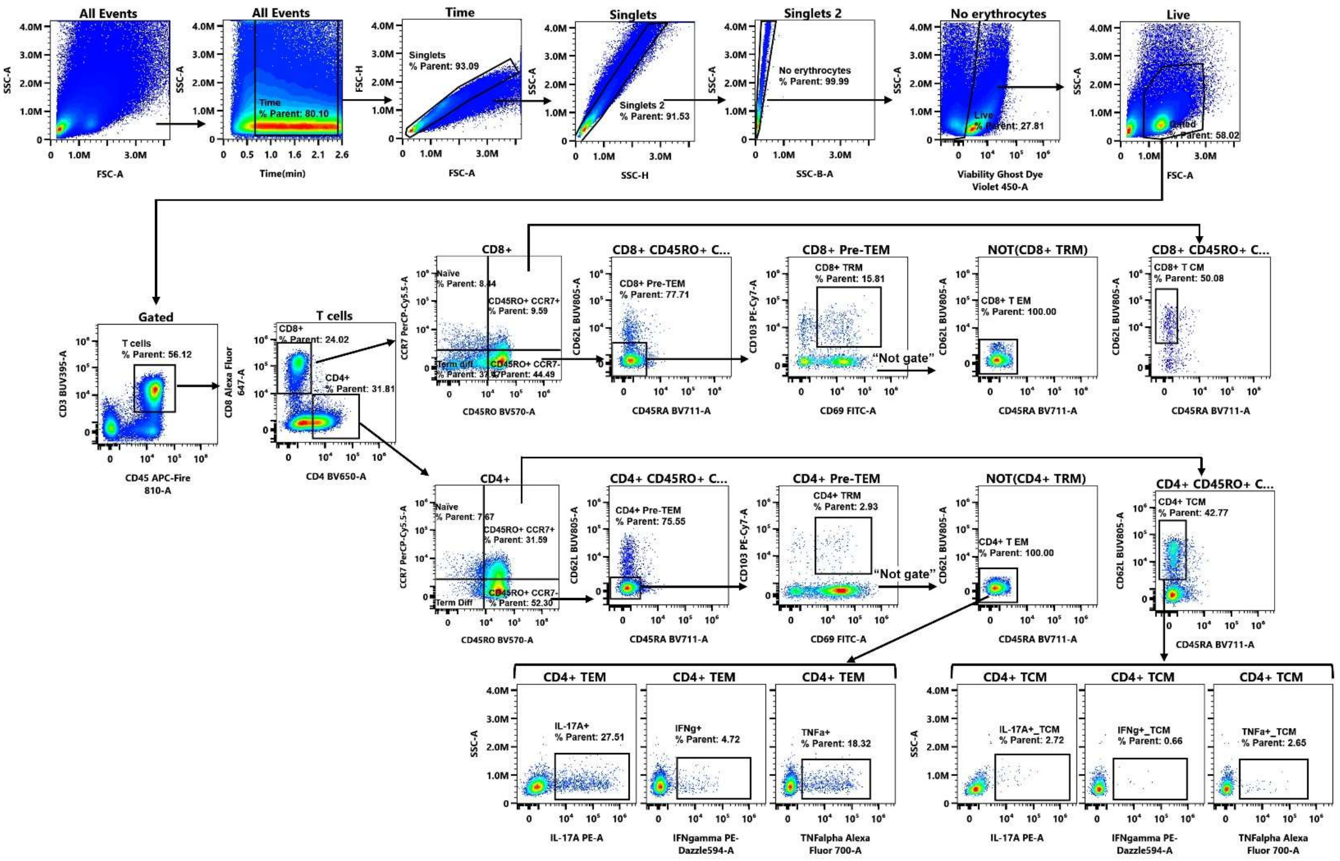

Supplementary Figure S1. Representative flow cytometry plots illustrating the hierarchical gating strategy used to identify T cell populations in skin and PBMCs. Initial events were gated on Time to ensure fluidic stability and exclude spurious events caused by flow rate fluctuations. Singlets were identified using FSC-A vs FSC-H and SSC-A vs SSC-H to exclude cell doublets. Erythrocytes were excluded by SSC-B-A (blue laser SSC-A) vs SSC-A. Live cells were gated as Ghost Dye Violet 450-negative cells, followed by exclusion of debris via FSC-A vs SSC-A. CD3+CD45+ total T cells were identified and further subdivided into CD4+ and CD8+ lineages. Within the CD4+ and CD8+ gates, memory populations were defined using CD45RO and CCD7 expression: naïve (CD45RO- CCR7+), terminally differentiated (Term diff, CD45RO- CCR7-), CD45RO+ CCR7+ cells were further gated to define central memory (T_{CM}) as CD45RA- CD62L+, while from CD45RO+ CCR7- cells, a "pre T_{EM}" population was isolated by selecting CD45RA- CD62L- cells. Within this gate, tissue-resident memory T cells (T_{RM}) were identified by the coexpression of CD103 and CD69. A boolean "NOT" gate was then applied to exclude T_{RM} events, yielding a purified conventional effector memory (T_{EM}) population. Representative plots show the gating for IL-17A+, IFN-γ+ and TNF-α+ populations within the memory compartments.
