# Supplementary material for: Reorganization and functional divergence of the CD4+ memory T cell compartment in hidradenitis suppurativa
Source: Front Immunol. 2026 Jul 14;17:1831664. doi: 10.3389/fimmu.2026.1831664 (PMC13407087; doi:10.3389/fimmu.2026.1831664)
Supplement: Supplementary file 2 [file DataSheet2.pdf]

Supplementary Table 1. Clinical information of hidradenitis suppurativa patients

Patient ID	Sex	Age (years)	Hurley Score	Smoking status	BMI (kg/m^2)
P01	F	42	II	Y	32
P02	F	55	II	N	25
P03	M	25	II	Y	22
P04	F	18	II	N	25
P21	F	52	II	N	23
P24	F	53	III	Y	30
P25	M	64	II	N	33
